# The Moderation of Human Characteristics in the Control Mechanisms of Rumours in Social Media: The Case of Food Rumours in China

**DOI:** 10.3389/fpsyg.2021.782313

**Published:** 2022-01-17

**Authors:** Sangluo Sun, Xiaowei Ge, Xiaowei Wen, Fernando Barrio, Ying Zhu, Jiali Liu

**Affiliations:** ^1^College of Economics and Management, South China Agricultural University, Guangzhou, China; ^2^Research Center for Green Development of Agriculture, South China Agricultural University, Beijing, China; ^3^School of Business and Management, Queen Marry University of London, London, United Kingdom

**Keywords:** food rumours, social media, anti-rumours, rumour control, human characteristics food rumours, human characteristics

## Abstract

Social networks are widely used as a fast and ubiquitous information-sharing medium. The mass spread of food rumours has seriously invaded public’s healthy life and impacted food production. It can be argued that the government, companies, and the media have the responsibility to send true anti-rumour messages to reduce panic, and the risks involved in different forms of communication to the public have not been properly assessed. The manuscript develops an empirical analysis model from 683 food anti-rumour cases and 7,967 data of the users with top comments to test the influence of the strength of rumour/anti-rumour on rumour control. Furthermore, dividing the users into three categories, Leaders, Chatters, and General Public, and study the influence of human characteristics on the relationship between the strength of rumour/anti-rumour and rumour control by considering the different human characteristics as moderator variables. The results showed that anti-rumours have a significant positive impact on the control of rumours; the ambiguity of rumours has a significant negative impact on the Positive Comment Index (PCI) in rumour control. Further, the Leaders increased the overall level of PCI, but negatively adjusted the relationship between evidence and PCI; the Chatters and the General Public reduced the overall level of PCI, and Chatters weakened the relationship between the specific type of anti-rumour form and PCI while the General Public enhanced the relationship between the specific type of anti-rumour form and PCI. In the long run, the role of Leaders needs to be further improved, and the importance of the General Public is growing in the food rumour control process.

## Introduction

In December 2019, a new strain of a Coronavirus, now identified as the SARS-CoV-2, had been transmitted to humans and the Coronavirus disease COVID-19 started to become a potential global pandemic with over 14 million confirmed cases so far. According to the WHO, coronaviruses are zoonotic viruses transmitted between animals and people, whose origin could be found somewhere in the food chain. The connection between food and the treatment of diseases has been widely analysed by academics and permeated the popular culture (Adelman and Haushofer, 2018). When a novel disease appears or starts to spread, a vast array of treatments based on food will be proposed. In the case of the novel COVID-19, several food-based treatment methods to control the disease, regardless of the scientific basis, have emerged with the lack of scientific understanding of the Coronavirus and the continuous media attention. The extensive use of social media by specialists and charlatans have contributed to the diffusion of those unscientific methods, leading to the creation and propagation of rumours. Facing the serious situation of novel coronavirus pneumonia and leaving aside the good or bad intentions of the originators, the propagation of rumours, such as dripping sesame oil into the nostrils, chewing garlic, drinking alcohol, and smoking vinegar can resist the virus, have made it more difficult to prevent and control the epidemic.

Rumours refer to messages that are typically conveyed by word of mouth (including digital word of mouth) and concerned about uncertain matters that have not been officially verified. The rumours are attractive because it seems to be new, important, and able to express emotion or satisfy some emotional needs such as anger and hatred (Knapp, 1944; Allport and Postman, 1947; Rosnow, 1991; Paek and Hove, 2019). In health and food risk research, rumours have been conceived as untrue narratives, misunderstandings, and obstacles to official prevention or management programs (Barrelet et al., 2013).

According to this, food rumours fall broadly into two categories. One is based on misunderstandings with the unconfirmed explanations about food, such as white eggs are more nutritious. The other is the fabricated one, with the deliberate fabrication of false food information, such as the plastic seaweed scandal that led to the detention of 18 people (Lin, 2017). It seems that consumers are generally uncertain about the safety and quality of their food, and their risk perception differs substantially from that of experts (Verbeke et al., 2007). In the case of China, many people have mistakenly expanded the scope of the medicine food homology, and simply believe that you are what you eat, such as the belief that eating a fish or a monkey’s brain is supposed to make people smarter. In the same way, people also often mistakenly believe that the meat and products of wild animals have certain therapeutic effects, such as Chinese pangolin meat helps alleviate rheumatism. With the cultural traits, the rumours will reinforce those mistaken beliefs sustainably. So, to avoid these misunderstandings, the current diet and health concepts and cognition need to be improved (Li et al., 2020).

Due to a number of food-related incidents continue to be reported frequently in China, consumer confidence has begun to vacillate (Jevsnik et al., 2008; Wen et al., 2019). Small risk can turn into a major food scare when the media repeatedly reports bad news about food hazards (Rutsaert et al., 2013; Ha et al., 2020). According to the data released by the Eighth China Food Safety Forum in 2016, food safety rumours have ranked first in the spread of online rumours, with a ratio of 45%. China Health Media Group released the “2017 Food Rumours Governance Report,” showing that the top five food rumours are about fruits, meat products, aquatic products, grain, and vegetables. From the perspective of communication channels, Weibo, a Chinese platform similar to Twitter, has become the main platform to spread food rumours, and small videos have become an important form to spread the rumours of food. The short video about the rumour of “Plastic seaweed” with only 137 s caused a loss of nearly ¥100 million to the seaweed industry in Fujian Province. To prevent or at least mitigate such harms, it is critical to establish a mechanism to control risk-prone false rumours promptly and effectively.

In issues related to health and food, communication with the public is a key component of the quality risk assessment process. Consumers need to know which behaviours are most likely to result in illness to make correct decisions about food handling and consumption behaviours (Hillers et al., 2003; Jevsnik et al., 2008). When people are not directly influenced by risks, they acquire risk information and develop risk perceptions through some form of communication, like word of mouth, mass media, or social media. The rapid growth of social media and new media have changed the method of people to get risk-related information. The public at large actively produce, seek, and share information instead of receiving it passively. With this context, other stakeholders may contribute to the widespread of inaccurate and false rumours, causing panic, health risks, and economic losses, and undermining the risk communication efforts of the regulator (DiFonzo and Bordia, 2007; Kaler, 2009; Xiao et al., 2019).

Social media has become a powerful communication medium as it is highly accessible due to its low cost and user friendliness (Veil et al., 2011). The use of blogs and other forms of social media may show that it is open to discussing issues rather than one way communication. It is important for the regulatory agencies to participate more actively in the risk communication on social media because consumers seem to be much keener on receiving and sharing negative news than positive news, and a false story spreads six times faster than a real story (Vosoughi et al., 2018). Compared to those bringing positive news about food safety issues, institutions or individuals who are prepared to produce negative news (e.g., the relationship between hormone residues and cancer or the impact of antibiotics in livestock production on human antibiotic resistance) are more likely to spread in the public through mass media, with a far greater and faster impact on consumer behaviour (Verbeke and Ward, 2001; Verbeke et al., 2007). This means that controlling the rumours on social media and promoting the dissemination of anti-rumours is not only relied on algorithms but also based on the consideration of user psychology.

The early control method of network rumour was to delete nodes, and the effectiveness of this method was confirmed in many experiments (Kimura et al., 2009), but the effectiveness of rumour management depends not only on how the anti-rumours respond to rumours but also on how people evaluate and react to the anti-rumours. A key factor influencing these public reactions is personal characteristics. Furthermore, consumers are individuals with highly variable psychological, attitudinal, and cultural characteristics, which cause them to react in a specific manner when facing food or lifestyle-related hazards. A successful food anti-rumour can convey food risk information to consumers (Dundes and Rajapaksa, 2001), and therefore risk communication should be based on consumer risk perceptions, concerns, information needs, and preferences, rather than expert-focused technical risk assessments (Cope et al., 2010; Charlebois and Summan, 2015; Vainio et al., 2020).

To study the influence of the strength of rumour/anti-rumour on rumour control by considering the different human characteristics as moderator variables, this manuscript investigates the relationships among the anti-rumour effect and the strength of rumour/anti-rumour, applying a structural equation modelling (SEM) to reveal such relationships.

## Conceptual Framework

**FIGURE 1:**
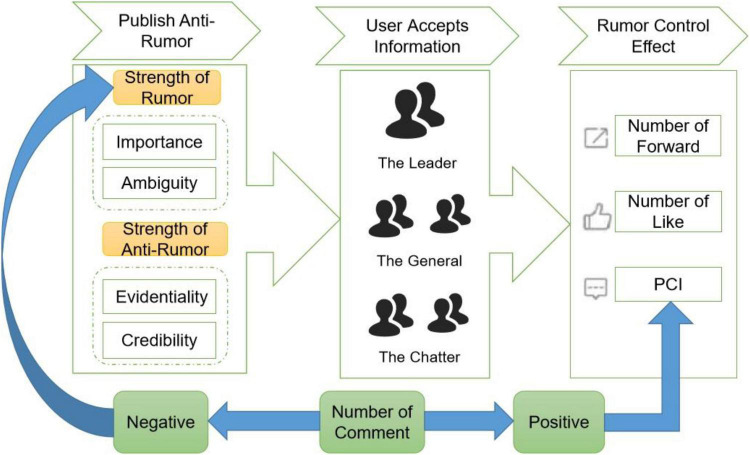
Rumour control process based on releasing anti-rumour.

The strategy to control the propagation of rumours is to send anti-rumours, which is an effective and safe way to correct false risk rumours (Paek and Hove, 2019). Anti-rumours are usually issued by a rumour control centre which is an official/unofficial organization or individual responsible for verifying the authenticity and responding to the news in its relevant field (Askarizadeh et al., 2019). Based on the rumour propagation and disintegration model of Zhang et al. (2014), we divide the rumour-breaking process into three stages: first, the rumour control centre releases the rumour-breaking information; second, the consumer accepts the anti-rumour information; third, the consumer’s reaction and the rumour control effect are observed (Figure 1).

We use the number of a repost (NR), the number of likes (NL), and the Positive Comment Index (PCI) as dependent variables to measure the rumour control effects. The PCI derives from Eq. 1. *N*_*NC*_ and *N*_*PC*_ are the number of negative and positive comments, respectively, and *N* is the total number of popular comments. In addition, the strength of anti-rumour (SAR), the credibility (CR) of the rumour control centre (CR), the evidentiality of the content (EV), the form of anti-rumour (FAR), the strength of rumour (SR), the importance of rumour (IM) subject, the ambiguity of rumour (AM) information, and the personal characteristics are assumed to influence the rumour control effects as the independent variables:


(1) $$PCI=\frac{N_{NC}-N_{PC}}{N}$$

### The Impact of the Strength of Anti-rumour on the Rumour Control

The strength of anti-rumour (SAR) is directly proportional to the CR of the rumour control centre and the evidentiality (EV) of the content. The anti-rumour will be more easily believed when the rumour control centre is confident to prove it with evidence (Askarizadeh et al., 2019). The strength of anti-rumour is obtained by using formula (2):


(2) SAR=EV×CR,0≤EV≤1,0≤CR≤1

Even with well-designed anti-rumours, they cannot achieve intended results if people distrust their information sources. CR is the unification of the public’s acceptance and trust in information promulgator, which consists of expertise (sometimes referred to as knowledge or competence) and trustworthiness (Metlay, 1999; Frewer et al., 2003). What the CR measures is the influence of the rumour control centre. The greater the number of fans or followers, the stronger the CR. We assign a CR to a number between (0, 1) based on the number of fans as shown in Table 1. In addition, evidentiality is based on the type of evidence provided by the author. Based on the study by Askarizadeh et al. (2019), Table 2 shows the EV value (0, 1).

**TABLE 1:** The assigned degree of credibility of rumour control centre (CR).

Credibility	Degree
>50 million	0.9
10–50 million	0.7
5–10 million	0.5
1–5 million	0.3
<1 million	0.1

**TABLE 2:** The assigned degree of evidentiality of anti-rumour (EV).

Evidentiality	Degree
First-hand experience	0.9
URL pointing to evidence	0.8
Quotation of person/organization	0.7
Attachment of picture	0.4
Quotation of unverifiable source	0.3
Employment of reasoning	0.2
No evidence	0.1

In addition, we also consider the influence of the FAR on the control effect of food rumour. The FAR is divided into two categories, one is the single type, that is, anti-rumour information for individual rumours; the other is the collection type, that is, for multiple rumours. The two types are recorded as 1, 0, respectively. The list of symbols is given in Table 3.

**TABLE 3:** List of symbols.

Variable	Descriptions
SAR	Strength of anti-rumour
SR	Strength of rumour
RC	Rumour control
PCI	Positive comment index
NR	Number of repost
NL	Number of likes
FAR	Form of anti-rumour

Therefore, the following hypotheses are proposed:

**Hypothesis 1a:** The evidentiality of anti-rumour has a significant positive impact on PCI.
**Hypothesis 1b:** The evidentiality of anti-rumour has a significant positive impact on the NR.
**Hypothesis 1c:** The evidentiality of anti-rumour has a significant positive impact on the NL.
**Hypothesis 2a:** The credibility of the announcer has a significant positive impact on the PCI.
**Hypothesis 2b:** The credibility of the announcer has a significant positive impact on the NR.
**Hypothesis 2c:** The credibility of the announcer has a significant positive impact on the NL.
**Hypothesis 3a:** The FAR has a significant positive impact on PCI.
**Hypothesis 3b:** The FAR has a significant positive impact on the NR.
**Hypothesis 3c:** The FAR has a significant positive impact on the NL.

### The Impact of Strength of Rumour on the Rumour Control

As shown in Eq. 3, the SR is proportional to the IM subject multiplied by AM :


(3) SR=IM×AM

The importance of food rumour depends on the severity (SV) of the rumour information (how much the rumour threatens people’s health) and the coverage (CV) of rumour (how many people are involved in a rumour; a minority, a region, or a country). According to the difference between CV and SV values, the importance can be calculated by Eq. 4 (Askarizadeh et al., 2019). For example, there is a high degree of severity and high coverage of rumour about overnight water being carcinogenic, while the rumour about the banana vinegar as a weight loss inducer has a low degree of severity and a low degree of coverage.


(4) $$IM=\sqrt{SV^{2}+CV^{2}},0\leq SV\leq 0.7,0\leq CV\leq 0.7$$

The ambiguity of food rumours reflects the misleading degree. There is no doubt that the false news is well-founded (Vosoughi et al., 2018). This article divides the AMs into the following six levels: First, the rumours derived from food safety incidents and the rumours containing real information have the highest ambiguity, such as the “poisoned bean sprouts” incident. Once the food safety incident is reported by the media, consumers have strengthened their sense of self-protection and will avoid buying all this type of food (Jevsnik et al., 2008). Second, unconfirmed misunderstanding of rumours, such as “tomatoes and crabs cannot be eaten together,” etc. This type of information has not been verified by scientific experiments but based on people’s experience and word of mouth for many years, they are also of high ambiguity. Compared with fabricated food rumours, misunderstanding of rumours are vaguer. Third, a large number of rumours will quote the opinions of other organizations or individuals to make the public easier to believe, such as “Heilongjiang Soybean Association said genetically modified soybeans cause cancer.” Fourth, the false information to be confirmed is the rumour that can be inferred based on common sense but has not been officially recognized as false information yet. Fifth, false promotion information are rumours caused by businesses to exaggerate the efficacy of their products, such as “enzymes can make people slim down and whiten,” and so on. Finally, the rumours that can be judged easily according to common sense and are officially recognized as false information have the lowest ambiguity. Take the “cotton floss” rumour as an example, cotton is an insoluble plant fibre whose taste is very different from floss. The person who spread this rumour has been administratively detained by the public security organ for 7 days in accordance with the law. In summary, according to the type of misinformation contained in rumours, the ambiguity (AM) is assigned a number between (0, 1), as shown in Table 4.

**TABLE 4:** The assigned degree of ambiguity of anti-rumour (AM).

Ambiguity	Degree
Somewhat certain	0.9
Derivative information on food safety incidents	0.8
Unconfirmed misunderstanding	0.7
Quotation of person/organization	0.6
False information to be confirmed	0.5
Exaggerated advertising information	0.4
Confirmed false information	0.3

Based on the aforementioned discussions, the following hypotheses are proposed:

**Hypothesis 4a:** The IM has a significant negative impact on PCI.
**Hypothesis 4b:** The IM has a significant negative impact on the NR.
**Hypothesis 4c:** The IM has a significant negative impact on the NL.
**Hypothesis 5a:** The AM has a significant negative impact on the number of PCI.
**Hypothesis 5b:** The AM has a significant negative impact on the NR.
**Hypothesis 5c:** The AM has a significant negative impact on the NL.

### Moderation of Rumour Control by Consumer Characteristics

Effective rumour management depends not only on how the anti-rumours respond to rumours but also on how people evaluate and react to the anti-rumours. A key factor that influences these public reactions are the personal characteristics of the consumers, but few studies explored the possible mediating role of consumer characteristic in the rumour control effect.

In this manuscript, consumer characteristics are divided into three categories: the Leader, the Chatter, and the General Public. Leaders are usually celebrities, their comments or information are more influential than usual users, they are more willing to spend more time investigating and thinking about events; the number of their followers exceeds largely the number of users they follow. Chatters are users who like to comment or send messages at will, who tend to participate in discussions actively; generally, the number of users they follow is larger than their followers. General Public is a category that consists of people that may stay in a specific group, who are not willing to communicate with people outside the group. They comment or send messages rarely and send comments or messages after consideration; the number of their followers and the number of users they follow tend to stay in balance. Any social media user that is not a Leader or a Chatter, is considered as General Public. Leaders can control the spread of rumours with their influence, but in a normal social situation, the General Public can also control rumours under certain conditions (Askarizadeh et al., 2019), all of which lead us to propose the last group of hypotheses:

**Hypothesis 6a:** The Leader positively moderates the relationship between evidentiality and PCI.
**Hypothesis 6b:** The Chatter negatively moderates the relationship between evidentiality and PCI.
**Hypothesis 6c:** The General Public positively moderate the relationship between evidentiality and PCI.
**Hypothesis 7a:** The Leader positively moderates the relationship between the FAR and PCI.
**Hypothesis 7b:** The Chatter negatively moderates the relationship between the FAR and PCI.
**Hypothesis 7c:** The General Public positively moderate the relationship between the FAR and PCI.

**FIGURE 2:**
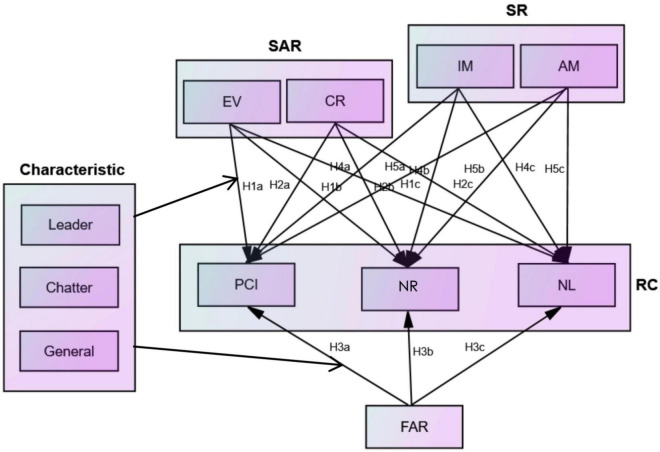
Theoretical model.

In summary, Figure 2 shows the theoretical model of this article and Table 5 shows the measurement of variables.

**TABLE 5:** Measurement of variables.

Variable	Measurement
CR	The number of fans (>50 million = 0.9; 10–50 million = 0.7; 5–10 million = 0.5; 1–5 million = 0.3; <1 million = 0.1
EV	First-hand experience = 0.9; URL pointing to evidence = 0.8; Quotation of person/organization = 0.7; Attachment of picture = 0.4; Quotation of unverifiable source = 0.3; Employment of reasoning = 0.2; No evidence = 0.1
SAR	*SAR* = *EV* × *CR*
IM	$IM=\sqrt{SV^{2}+CV^{2}},0\leq SV\leq 0.7,0\leq CV\leq 0.7$
AM	Somewhat certain = 0.9; Derivative information on food safety incidents = 0.8; Unconfirmed misunderstanding = 0.7; Quotation of person/organization = 0.6; False information to be confirmed = 0.5; Exaggerated advertising information = 0.4; Confirmed false information = 0.3
SR	*SR* = *IM* × *AM*
NNC	The number of negative comments
NPC	The number of positive comments
*N*	The total number of popular comments
PCI	$PCI=\frac{N_{NC}-N_{PC}}{N}$
NR	Number of repost
NL	Number of likes
FAR	The single type = 1; the collection type = 0

## Data Collection and Research Samples

We collected 683 food rumours identified by the rumour control centre on the Weibo platform in 2012–2018, along with 7,967 hot comment user’s data. The hot comments are the comments that attract more than 10 appraisals, which in Weibo are represented by a thumbs up image and implies a “like.” The more comments are liked, the hotter the comments are. Through the view of hot comment, it is judged whether people’s attitude toward anti-rumours is positive or not, whether it achieves the effect of rumour control, or the negative cause the rumours to spread again. Compared with the data from questionnaires and surveys, the data in this manuscript are the real traces left by people on the social network platform, avoiding the deviation caused by subjective suggestions with reliability and validity.

Table 6 shows the analysis of the data. Judging from the rumour control centre, “People’s Daily” and “CCTV News” are comprehensive media managed by government agencies that send a large number of anti-rumours, and both with a large number of followers. “Husk” and “Rumour shredder,” on the other hand, are professional media managed by non-government organizations, with a relatively smaller number of followers, and with also a relatively lower number of anti-rumours sent. In relation to the different types or participants, the Leader is the one with a smaller number of comments made, followed by the General Public, and then the Chatters.

**TABLE 6:** Sample feature analysis.

Item	Category	Amount	Percent
Food type	Fruits	141	20%
	Drink	83	12%
	Aquatic products	76	11%
	Meat	57	8%
	Vegetables	44	6%
Rumour control centre (number of fans)	People’s Daily (98.13 million)	315	47%
	CCTV News (89.02 million)	203	29%
	Husk (9.31 million)	99	14%
	Rumours shredder (1.46 million)	66	10%
Hot comment user characteristic	Leader	1,464	18.37%
	Chatter	3,309	41.53%
	General	3,194	40.09%
Hot comment user gender	Male	4,064	51.01%
	Female	3,308	41.52%
	Official Weibo	595	7.47%
Hot comment user qualifications	University and above	1,597	21.05%
	Unknown	6,370	79.95%
			

**FIGURE 3:**
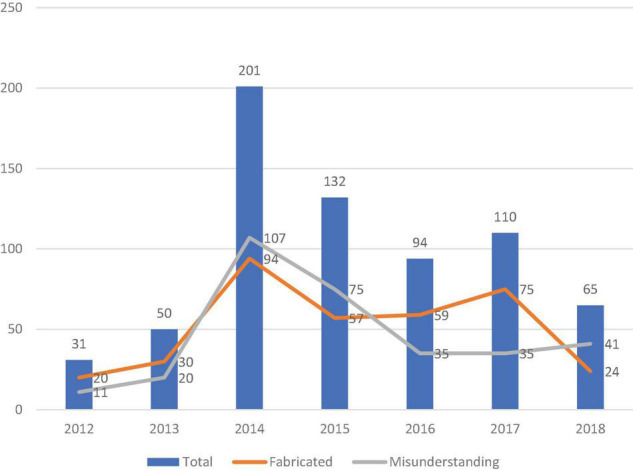
Food rumour trends.

**FIGURE 4:**
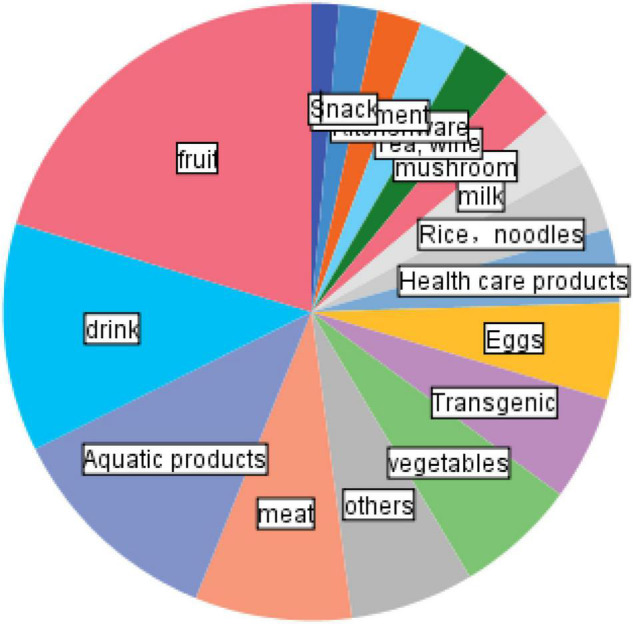
Food rumour classification.

Food rumours are developmental. It can be seen from Figure 3 that while fabricated rumours declined, the number of misunderstanding rumours showed an upward trend, which means consumers have improved their ability to screen out deliberately fabricated rumours of food, but misunderstandings about food nutrition and health still prevail. With the continuous improvement of people’s living standards, the upgrading of dietary structure, and the increasing acceptance of food safety standards by consumers, issues that seem to be safe within the scope of the original standards need to be redefined, such as the debate about the impact of vitamin supplements on the human body and the issue of food compatibility and many more. Misunderstanding food rumours are the focus of future food safety and health management risk communication. Food rumours are repetitive. According to statistics, about 70% of food safety rumours are rumours that have appeared before. From Figure 4 and Table 6, it can be seen that the top five food safety rumours are fruits, beverages, aquatic products, meat, and vegetables, accounting for 20, 12, 11, 8, and 6%, respectively. All are fresh agricultural products. Many food rumours can be spread again after changing the time and place. For example, rumours about melons and fruits will appear again every summer. For another example, a series of terms such as “water injection,” and “hormone injection” will be used in various foods. Food rumours are realistic. Most rumours originate from food safety incidents in real life. For example, in the “poisonous bean sprouts” incident in September 2013, criminals were found to use AB powder and rootless bean sprouts to process toxic and harmful “poisonous bean sprouts” and sell for profit. After the criminals were investigated and convicted, rumours about “poisonous bean sprouts” were still circulating on the Internet for a long time. Human-derived food safety incidents pose a major threat to public social health and greatly affect the public’s trust in the food industry. The public has doubts about the government, authoritative media whether they conceal the truth for fear of causing social panic. Food rumours are provocative. Judging from the text characteristics of the rumours, more than a quarter (26.94%) of the rumours encoded in the database of this article contain death arousal, such as words such as “carcinogenic,” “toxic,” “death,” and “harmful,” which easily trigger readers’ panic. Due to the lack of transparency in the current food supply chain, especially in the upstream part where most adulteration occurs. The public cannot obtain accurate food safety information in a timely manner. Food rumours are newsworthy to the public who are desperately pursuing the unknown, and satisfy the public’s curiosity and curiosity.

## Empirical Analysis and Hypothesis Testing

### Descriptive Statistical Analysis and Correlation Analysis

The mean, standard deviation, and correlation coefficient (Pearson correlation) of evidentiality, credibility, importance, ambiguity, the proportion of Leaders, the proportion of Chatters, the proportion of General Public, and demographic variables are shown in Table 7. Combining the mean and standard deviation of each variable, the influence of strength of rumour/anti-rumour on rumour control, and the moderation of human characteristics are analysed.

**TABLE 7:** Mean, SD, and correlation coefficient (*N* = 683).

	NR	NL	PCI	IM	*AM*	EV	CR	FAR	Leader	General	Chatter	Male	Female	Edu	OW
NR	1														
NL	0.444**	1													
PCI	0.024	–0.002	1												
IM	−0.199**	−0.081*	–0.058	1											
AM	0.112**	0.001	−0.110**	0.126**	1										
EV	–0.019	0.078*	0.344**	–0.033	−0.096*	1									
CR	0.041	0.382**	−0.081*	–0.010	–0.041	–0.045	1								
FAR	−0.182**	−0.222**	0.127**	0.120**	0.006	0.154**	−0.278**	1							
Leader	0.028	0.040	0.315**	–0.002	−0.195**	0.096*	0.234**	−0.103**	1						
General	−0.164**	−0.185**	−0.131**	0.139**	–0.036	−0.114**	−0.216**	0.070	−0.338**	1					
Chatter	0.157**	0.213**	−0.113**	−0.143**	0.178**	0.044	0.053	–0.008	−0.387**	−0.726**	1				
Male	–0.079	–0.029	−0.095*	0.090*	0.061	0.091*	−0.263**	0.030	−0.079*	0.065	–0.004	1			
Female	0.147**	0.050	–0.043	–0.028	–0.015	−0.105**	0.183**	–0.021	−0.163**	0.084*	0.043	−0.796**	1		
Edu	−0.153**	−0.093*	0.043	0.023	0.051	0.016	−0.233**	0.067	−0.230**	0.244**	−0.077*	0.348**	−0.252**	1	
OW	–0.055	–0.002	0.255**	–0.061	–0.047	–0.028	0.233**	–0.065	0.428**	−0.294**	–0.029	−0.416**	−0.091*	−0.198**	1
Ave	5,690.27	1,402.15	0.313337	0.6380	0.615	0.680	0.783	0.15	18.4522%	39.7547%	42.2728%	0.5090	0.4103	0.2029	0.6858
Ste	10,038.339	1,606.015	0.4279588	0.19781	0.1797	0.2151	0.2137	0.355	13.0647%	17.3492%	17.7969%	0.1840	0.1657	0.1407	0.9639

*Significant at 10%, **significant at 5%, ***significant at 1%.

The correlation coefficient is shown in Table 7. First, the importance of food rumour is significantly negatively correlated with the number of Reposts in the rumour control (*r* = –0.199, *p* < 0.01), the ambiguity of food rumours is significantly positively correlated with the number of Repost in rumour control (*r* = 0.112, *p* < 0.01), the single FAR is significantly negatively correlated with the number of Repost in rumour control (*r* = –0.182, *p* < 0.01). The importance of food rumours and the number of Likes in rumour control are significantly negatively correlated (*r* = –0.081, *p* < 0.05), the evidentiality of anti-rumours and the number of Likes in the rumour control are significantly positive correlation (*r* = 0.078, *p* < 0.001). The AMs and the positive commentary index in the rumour control are significantly negatively correlated (*r* = –0.110, *p* < 0.01), the evidentiality of anti-rumour and PCI in rumour control are significantly positive correlation (*r* = 0.344, *p* < 0.01), the credibility of the rumour control centre and the PCI in the rumour control are significantly negatively correlated (*r* = –0.081, *p* < 0.05), the single FAR and the PCI in rumour control are significantly positively correlated (*r* = 0.127, *p* < 0.01).

Second, the proportion of Leaders in users is significantly positively correlated with the PCI in the rumour control (*r* = 0.315, *p* < 0.01); the proportion of General Public in users and the number of Reposts, the number of Likes and the PCI in the rumour control (*r* = –0.164, –0.185, –0.131, *p* < 0.01) are significantly negatively correlated; the proportion of Chatters in users and the number of Reposts as well as the number of Likes in the rumour control (*r* = 0.157, *r* = 0.213, *p* < 0.01) are all significantly positively correlated, while the proportion of Chatters in the user group are significantly negatively correlated with the PCI (*r* = –0.113, *p* < 0.01); these are consistent with the theoretical model expectations, The model assumption provides initial support.

### Hypothetical Test

We use Amos24 to construct the structural equation model and to explore the relationship between IM, AM, evidentiality of anti-rumour, the credibility of rumour control centre and rumour control measures, and possible mechanisms of interaction. We test the research hypothesis in turn. The path coefficient and the significant result of the strength of rumour/anti-rumour to the rumour control are shown in Table 8. Table 9 shows the adaptability of the model. It can be seen that the index value reached the ideal level in this study, indicating that the model’s adaptation effect is generally good. (1) The strength of anti-rumours plays a partial positive role in the control of food rumours. Among them, the evidentiality of anti-rumours has played a positive role in the NR and the NL and the PCI, and the credibility of the rumour control centre has played a positive role in the NL. This indicates that the stronger the evidence of anti-rumour, the better the rumour control effect; the stronger the credibility of the rumour control centre, the higher the user’s approval of anti-rumour. The standardization path coefficient of the CR and NR, as well as PCI, are –0.035, –0.054, *P* > 0.1, which indicates the credibility of the rumour control centre has no significant effect on the user’s proliferation and positive comments. H1a, H1b, H1c, and H2c are supported.

**TABLE 8:** Significance test of model path coefficients.

			Estimate	C.R.	*P*	Test result
NR	<—	EV	0.066	1.752	0.080	*
NR	<—	CR	–0.035	–0.908	0.364	Ns
NR	<—	FAR	–0.174	–4.465	***	***
NR	<—	IM	–0.181	–4.852	***	***
NR	<—	AM	0.122	3.276	0.001	***
NL	<—	EV	0.088	2.797	0.005	***
NL	<—	CR	0.366	11.427	***	***
NL	<—	FAR	–0.059	–1.788	0.074	*
NL	<—	IM	0.018	0.561	0.575	Ns
NL	<—	AM	–0.019	–0.608	0.543	Ns
PCI	<—	EV	0.321	8.797	***	***
PCI	<—	CR	–0.054	–1.369	0.171	Ns
PCI	<—	FAR	0.069	1.821	0.069	*
PCI	<—	IM	–0.046	–1.292	0.196	Ns
PCI	<—	AM	–0.076	–2.101	0.036	**

*Significant at 10%, **significant at 5%, and ***significant at 1%.

**TABLE 9:** Model fitting index.

Fitting index	CMIN/DF	*P*	CFI	IFI	NFI	RMSEA
Suggestive value	1–3	<0.05	>0.90	>0.90	<0.05	
Actual value	2.514	0.028	0.985	0.986	0.977	0.047
Fitting effect	Accepted	Accepted	Accepted	Accepted	Accepted	Accepted

(2) The FAR plays a partial positive role in the control of food rumours. The standardized path coefficients of the single form for the NR and the NL are –174, *P* < 0.01; –0.059, *P* < 0.1, which indicates that the single FAR has a negative impact on the NR and the NL. The standardized path coefficient of the single form for the PCI is 0.069, *P* < 0.1, which indicates that single-form anti-rumour has a significant effect on positive comments of users. H3a is supported.

(3) The strength of food rumour plays a partial negative role in rumour control. Among them, the IMs played a negative role in the NR, and the AMs played a negative role in the PCI. The standardized path coefficients of rumour importance and ambiguity for the NL are 0.018, –0.019, *P* > 0.1, which indicates that the importance and AMs have no significant effect on the NL. The standardized path coefficient of rumour importance for the PCI is –0.046, *P* > 0.1, which indicates that the influence of rumours importance on the PCI is not significant. H4b and H5a are supported.

### Moderation Effect Test

We further explore how human characteristics influence people’s decisions to send rumour/anti-rumours. Therefore, the characteristics are used as a moderating variable to test its moderating effect on the positive review index. To avoid the problem of multiple collinearities between independent variables, dependent variables and moderate variables, the independent variables and moderate variable data are centralized. The independent variables, the moderate variables, and the interactions between the independent variables and the moderate variables are sequentially subjected to regression, and the resulting variables are predicted. The results are shown in Table 10.

**TABLE 10:** Test results of human characteristics moderate effect (*N* = 683).

Item	Variables	Positive comment index (PCI)		
		Model 1	Model 2	Model 3
Independent variables	EV	0.410***		
	FAR		0.124***	0.119***
Moderate variables	Leader	0.288***		
	General			–0.134***
	Chatter		–0.118***	
Interaction term	EV × L	–0.118**		
	FAR × G		–0.102***	
	FAR × C			0.116***
	*R* ^2^	0.204	0.039	0.049
	Δ*F*	4.376	7.285	9.443
	Δ*R*^2^	0.005	0.010	0.013

*Significant at 10%, **significant at 5%, and ***significant at 1%.

According to Model 1, when the interaction items of evidentiality and Leader percent, as well as evidentiality and Leader percent enter the regression equation in turn, the interaction term of the evidentiality and the Leader percent has a significant influence on the PCI (β = –0.118, *p* < 0.05), indicating the relationship between the evidentiality and the PCI is negatively moderated by the Leader, H6a is opposite. According to Model 2, the interaction term of the and FAR and Chatter has a significant influence on the PCI (β = –0.102, *p* < 0.01), indicating the relationship between the FAR and PCI is negatively moderated by the Chatter, H6c is supported. According to Model 3, the interaction term between the FAR and General Public has a significant influence on the PCI (β = 0.116, *p* < 0.01) is, indicating that the General Public have a positive moderate effect on the relationship between FAR and PCI, H6d is opposite.

**FIGURE 5:**
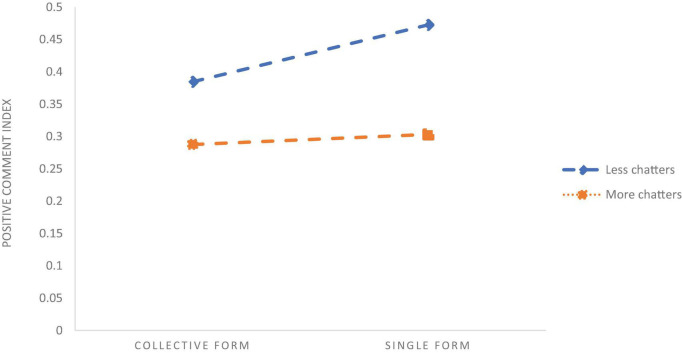
Chatter’s role in moderating the form of anti-rumour.

**FIGURE 6:**
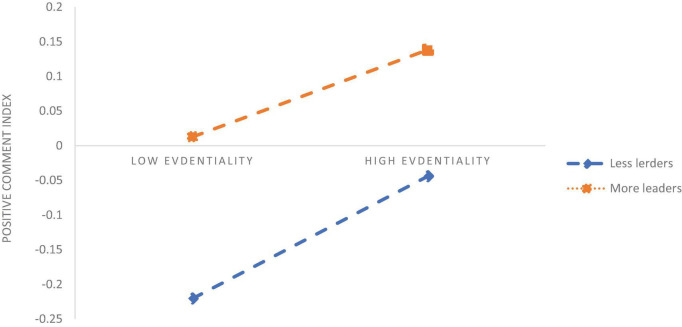
Leader’s role in moderating the evidentiality.

**FIGURE 7:**
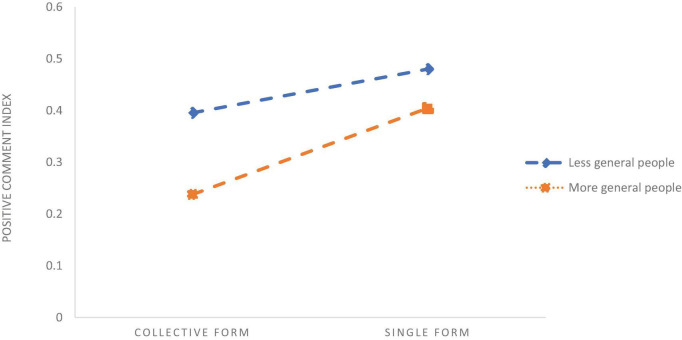
General Publics’ role in moderating the form of anti-rumour.

To analyse how the interaction items of evidentiality, FAR and percent of Leaders, Chatters, General Public affect the PCI it is necessary to further test the direction of moderate effects. Therefore, we draw figures of the effects of the Leader, the Chatter, and the General Public on the relationship between evidentiality, FAR, and PCI (Figures 5–7).

Figure 6 shows that the overall PCI is at a higher level in the case of a high percentage of Leaders in the population than the low Leader percentage, but the positive impact of evidentiality on the PCI is reduced. Figure 5 shows that the overall PCI is at a higher level in the case of a lower Chatter percent. With more Chatters in the population, the positive impact of the single FAR on the PCI has reduced significantly. Figure 7 shows that the overall PCI is at a lower level in the case of the more General Public, but the positive impact of the single FAR on the PCI is significantly increased. Relative to the low Leader ratio, the Leaders in the crowd raised the overall positive comments index, and the Chatters and the General Public reduced the overall positive comment index. However, as the number of Leaders in the population increases, the positive impact of the evidentiality on the positive comment index is diminished; with the entry of Chatters, the positive impact of single form on the PCI is significantly weakened; with the increase of the General Public, the positive impact of the single form on the PCI is significantly enhanced.

## Discussion

About 683 food rumours are officially identified in 2012–2018, and they are used as investigation cases to empirically test the influence of the strength of anti-rumour (evidentiality, credibility), the SR (importance, ambiguity), and the anti-rumour form on rumour control, with the human characteristics as the moderate variable. The results are as follow: First, among the strength of anti-rumour, evidentiality has a significant positive influence on rumour control, which has the greatest impact on the positive comment index, followed by the number of Reposting, and again the number of Likes. Credibility has a significant positive impact on the number of Likes; there is no significant impact on the number of Reposts and the positive comment index. Second, among the SR, ambiguity has a significant positive impact on the number of Repost and negative impact on the positive comments index and has no significant effect on the number of Likes: importance has a significant negative impact on the number of Reposts, and has no significant effect on the number of Likes and the positive comment index. Third, the single FAR has a significant negative impact on the number of Reposts and the number of Likes and has a significant positive effect on the positive commentary index. Fourth, the Leader’s negative moderating on the relationship between evidentiality and positive comments index is significant; the Chatters negatively moderate the influence of single form on the positive commentary index; the General Public positively affect the influence of single form on the positive commentary index.

## Conclusion

The manuscript analyses the diffusion and control of food rumours on social media, which becomes a popular topic due to the COVID-19 pandemic that originated in a food-related virus. Food rumours have been widely studied and been deemed to be misunderstood and fabricated. Regardless of the type of rumour, due to their potential impact on public health and the economy, it needs to be controlled and minimised. To properly control food rumours on social media, the psychological characteristics of the consumers need to be incorporated into the analysis and tested. The results with the incorporation of social network users’ psychological characteristics are as follows.

First of all, from the point of strength of anti-rumours, the evidentiality has significantly increased the number of Reposts, the number of Likes, and the positive comment index, which is the main driving force for food rumour control. With the continuous improvement of scientific literacy, the public pays more attention to rigorous experimental evidence and prudent conclusions instead of only listening to a simplistic take-or-leave conclusion. The credibility of the rumour control centre significantly increased the number of Likes but did not increase the number of Reposts and the positive comment index. The official Weibos such as the People’s Daily and CCTV News have strong credibility and a large fan base, so they have a high number of Likes. For food rumours, the public is more inclined to support professional science organizations such as Husks and Rumour shredder, even though they have relatively fewer fans. This finding implies that even government officials or risk managers still need to make efforts to establish communication and engage with the audience. Instead of simply refuting different voices, authorities and food related organisations should adopt scientific evidence-based anti-rumours to be more convincing.

Second, from the perspective of the FARs, the single FAR will reduce the number of Repost and Likes but have a positive impact on the positive comment index; the multiple FAR can increase the number of Repost and the number of Likes. However, it has a negative impact on the positive comment index. Taking into account the characteristics of these two forms of anti-rumours, the single anti-rumour is more eloquent, often accompanied by evidence links or videos, but with relatively low heat; the multiple anti-rumours with insufficient evidence are subject to space restrictions due to a large number of anti-rumours, involving a wide range of content and high heat. This also confirms that the evidentiality of anti-rumour is the key to controlling negative comments. Users who repost and likes the anti-rumour information pay more attention to the form rather than the content, and the users who make the comments pay more attention to the EV and then give support or objection.

Third, from the perspective of SR, the IMs has a negative impact on the number of Reposts. The AMs has a negative impact on the positive comment index. The importance and AMs both increase the difficulty of rumour control. However, the AMs has a positive impact on the number of Reposts. Considering the characteristics of ambiguity, the higher the AM, the more misleading it is, and the more difficult it is to refute rumours. The public tends to Repost the anti-rumours of higher ambiguity rumours to obtain more valuable information. However, while the number of anti-rumours Reposts has increased, the negative commentary information has also spread. Therefore, the fundamental control of food rumours is to control the negative comments. The strategy to effectively control negative reviews is not to block and delete, but to cultivate a group of users who can issue neutral comments as the “immune system” of social networks, thereby diluting different negative comments (Johnson et al., 2019).

Finally, the effects of human characteristics as a moderate variable on food rumour control were explored. We verify that Leaders have played an active role in the rumour control process. The population with a higher proportion of Leaders has more positive comments; however, as the proportion of Leaders increases, the relationship between evidentiality and positive comment index is moderate negatively, which is not conducive to the role of opinion Leaders. So, Leaders should pay more attention to the evidentiality of anti-rumour information, leading the public to further thinking. The increase in the number of Chatters and the General Public will reduce the number of positive comments; however, the General Public has enhanced the relationship between the anti-rumour form and the positive commentary index, which indicates that with the continues improvement of the General Public’s scientific literacy, more attention is paid to the content of information rather than form, gradually reduces the number of negative comments posted, and the General Public are becoming the backbone of the control of food rumours. The popularization of science and propaganda should be strengthened to advocate well-informed and healthy diet culture, while education on food safety should be carried out from early age.

Some limitations of the study need to be taken into account when interpreting the results and their contributions. First, this research focused on food rumours in a social media (Weibo) and in a country (China). Since consumers from different socioeconomic backgrounds may access the Internet in different ways, the Weibo data used in this study may only imply the participants belong to specific socioeconomic and cultural groups. In addition, other determinants such as risk perception, emotional response, and participation may also affect people’s attitudes toward rumours and anti-rumours, and the effectiveness of rumour control (Kuttschreuter, 2006). Future research considering these factors may refine the current findings. Constrained by various reasons, the model in this manuscript has not discussed the misunderstanding type and the fabricated food rumours separately. Due to their different characteristics and effects, consumer perceptions and attitudes are also quite different, the corresponding control strategies may be different. Therefore, future research can further refine the model. The theoretical model in this article is not only suitable for explaining the control mechanism of food rumours, but also may be suitable for analyzing the effect of public-facing risk communication. Future research can further test its effectiveness.

Therefore, improve the evidentiality of anti-rumour with first-hand materials such as experimental proof to improve the scientific credibility of the conclusion. In addition, due to the diversity and complexity of negative comments, different methods and strategies should be adopted together to reduce negative comments effectively and efficiently. Meanwhile, food safety supervision departments at all levels should establish and improve food safety emergency plans and mechanisms and establish special risk communication departments, keeping active in social media with a reputation to strengthen food safety risk communication. Finally, comprehensively promoting relative education is significant and essential. The public’s knowledge of “food” should be improved to enhance the public’s information discrimination ability, with a greater influence of leaders and publics on network consensus, finally establishing the long-term governance mechanism with government guidance, social co-governance, public participation, and legal safeguard.

## Data Availability Statement

The raw data supporting the conclusions of this article will be made available by the authors, without undue reservation.

## Author Contributions

SS, XG, and XW wrote the manuscript. YZ and JL analyzed the data. FB provided suggestions and revised the article. All authors contributed to the article and approved the submitted version.

## Conflict of Interest

The authors declare that the research was conducted in the absence of any commercial or financial relationships that could be construed as a potential conflict of interest. The reviewer QG declared a past collaboration with one of the authors XW.

## Publisher’s Note

All claims expressed in this article are solely those of the authors and do not necessarily represent those of their affiliated organizations, or those of the publisher, the editors and the reviewers. Any product that may be evaluated in this article, or claim that may be made by its manufacturer, is not guaranteed or endorsed by the publisher.
